# Exploring the mechanism of ellagic acid against gastric cancer based on bioinformatics analysis and network pharmacology

**DOI:** 10.1111/jcmm.17967

**Published:** 2023-10-04

**Authors:** Zhiyao Liu, Hailiang Huang, Ying Yu, Lingling Li, Xin Shi, Fangqi Wang

**Affiliations:** ^1^ Department of Rehabilitation Medicine Shandong University of Traditional Chinese Medicine Jinan China; ^2^ Innovative Institute of Chinese Medicine and Pharmacy Shandong University of Traditional Chinese Medicine Jinan China

**Keywords:** bioinformatics analysis, ellagic acid, gastric cancer, network pharmacology

## Abstract

Ellagic acid (EA) is a natural polyphenolic compound. Recent studies have shown that EA has potential anticancer properties against gastric cancer (GC). This study aims to reveal the potential targets and mechanisms of EA against GC. This study adopted methods of bioinformatics analysis and network pharmacology, including the weighted gene co‐expression network analysis (WGCNA), construction of protein–protein interaction (PPI) network, receiver operating characteristic (ROC) and Kaplan–Meier (KM) survival curve analysis, Gene Ontology (GO) function and Kyoto Encyclopedia of Genes and Genomes (KEGG) pathway enrichment analysis, molecular docking and molecular dynamics simulations (MDS). A total of 540 EA targets were obtained. Through WGCNA, we obtained a total of 2914 GC clinical module genes, combined with the disease database for screening, a total of 606 GC‐related targets and 79 intersection targets of EA and GC were obtained by constructing Venn diagram. PPI network was constructed to identify 14 core candidate targets; TP53, JUN, CASP3, HSP90AA1, VEGFA, HRAS, CDH1, MAPK3, CDKN1A, SRC, CYCS, BCL2L1 and CDK4 were identified as the key targets of EA regulation of GC by ROC and KM curve analysis. The enrichment analysis of GO and KEGG pathways of key targets was performed, and they were mainly enriched in p53 signalling pathway, PI3K‐Akt signalling pathway. The results of molecular docking and MDS showed that EA could effectively bind to 13 key targets to form stable protein–ligand complexes. This study revealed the key targets and molecular mechanisms of EA against GC and provided a theoretical basis for further study of the pharmacological mechanism of EA against GC.

## 1 INTRODUCTION

Gastric cancer (GC) is the fifth most common malignant tumour in the world; although the incidence of GC is currently declining slightly in many countries and regions, it is the third leading cause of cancer‐related death worldwide; with an estimated 768,793 deaths worldwide in 2020,^1^ GC remains a major health problem plaguing the world^2^; surgery, chemotherapy, radiotherapy and targeted drugs are currently the main treatment strategies for patients with GC,^3^ which have made great progress in the treatment of GC, but since most GC patients are diagnosed in the middle and late stages, coupled with metastasis and recurrence; furthermore, existing treatment strategies will have adverse reactions and various forms of drug resistance,^4^ resulting in poor prognosis of patients with advanced GC. Therefore, there is an urgent need to develop new anticancer drugs with anticancer activity and low side effects.

Ellagic acid (EA) is a naturally occurring polyphenolic compound found in abundance in many fruits and nuts, such as pomegranates, grapes and blueberries, and many medicinal plants, such as *Cornus officinalis*, *Raspberry* and *Gentiana scabra*,^5^, ^6^, ^7^ because of its anti‐inflammatory, antioxidant, anticancer and other activities,^8^, ^9^ and its low cost, it has attracted more and more researchers' attention in the treatment and prevention of cancer. EA has been reported that EA can induce pancreatic cancer cell apoptosis and reduce cell proliferation by inhibiting the transcription factor NF‐κB.^10^ The Wang study showed that EA can exert an anti‐angiogenic effect in breast cancer through the VEGFR‐2 signalling pathway,^11^ and it also prevented the development of cisplatin resistance in the epithelial ovarian cancer cell line.^12^ Recent studies have shown that EA inhibits acid‐enhanced GC cell migration and invasion by inhibiting the expression of multiple factors, such as COX1, COX2, c‐myc, snail and twist1.^13^ In addition, Cheshomi et al.^14^ found that EA can inhibit the proliferation and migration of human GC AGS cells, induce apoptosis and reduce the expression of inflammatory genes, and the tissue test results of in vivo experiments show that the compound has no side effects, and its safety has been confirmed. In addition, GC is closely related to the human pathogen *Helicobacter pylori* (*H. pylori*), which has been classified as a group I carcinogen by WHO^15^; interestingly, multiple studies have shown that EA has anti‐*H. pylori* activity and has great potential for the prevention and treatment of *H. pylori*.^16^, ^17^ From this, we speculate that EA has the potential to prevent and treat GC by regulating multiple targets, multiple pathways and multiple biological processes. However, the current research involves few related targets and pathways and has not been fully explored. The mechanism of EA's action on GC has not been fully revealed, and its pharmacological mechanism needs to be systematically analysed.

**FIGURE 1:**
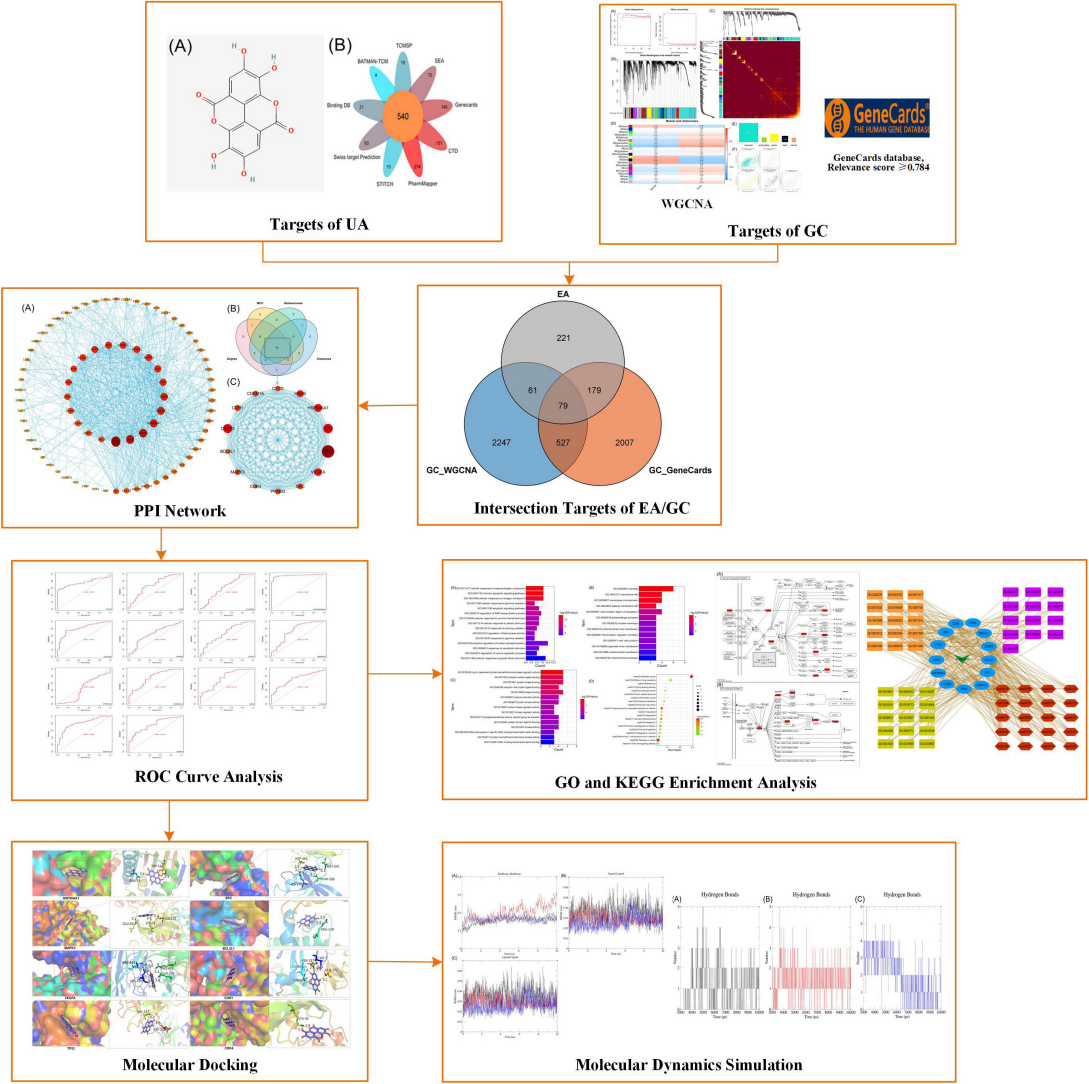
Flow chart of action mechanism of ellagic acid against gastric cancer.

Network pharmacology was first proposed by Hopkins in 2008^18^; it is a powerful method that combines systems biology, pharmacology, computer technology and other disciplines to predict the disease targets and molecular mechanisms of drug action.^19^, ^20^ Bioinformatics analysis is an important tool for identifying and evaluating genes associated with cancer development and progression^21^; weighted gene co‐expression network analysis (WGCNA) is a systems biology algorithm that identifies gene functions and the correlation between genes and clinical features, and identifies gene modules that are closely related to clinical features.^22^ Molecular docking is used to simulate and predict the binding mode and affinity between receptors and ligands,^23^ and molecular dynamics simulations (MDS) are based on Newtonian mechanics to simulate the motion of molecular systems in a system to evaluate the stability and flexibility of the system.^24^ In this study, network pharmacology and bioinformatics analysis combined with WGCNA, molecular docking and MDS were used to explore the target and potential mechanism of action of EA against GC. The flowchart of this study was shown in Figure 1.

## 2 MATERIALS AND METHODS

### 2.1 Screening of potential targets for EA

We obtained the ADME parameters of the EA from the TCMSP database (https://tcmsp‐e.com/tcmsp.php),^25^ including oral bioavailability (OB) and drug similarity (DL); the chemical information related to EA was obtained from the Pubchem database (https://pubchem.ncbi.nlm.nih.gov/),^26^ and then collected EA candidate targets from 9 databases, including TCMSP database, BATMAN‐TCM database (http://bionet.ncpsb.org.cn/batman‐tcm/),^27^ Binding DB (http://www.bindingdb.org/bind/index.jsp),^28^ SwissTargetPrediction web server (http://www.swisstargetprediction.ch/),^29^ STITCH database (http://stitch.embl.de/),^30^ pharmmapper web server (http://www.lilab‐ecust.cn/pharmmapper/index.html),^31^ CTD database (http://ctdbase.org/),^32^ GeneCards database (https://www.genecards.org/),^33^ SEA database (http://sea.bkslab.org/),^34^ combined with the UniProt database to standardize the names of the acquired targets,^35^ and then used the bioinformatics online platform (http://www.bioinformatics.com.cn/) to present each database to obtain the number of targets.

### 2.2 Screening of disease targets for GC and intersection targets for EA against GC

We obtained RNA‐Seq data and clinical data of TCGA‐STAD patients from the TCGA database (https://portal.gdc.cancer.gov/), using Perl script for preprocessing to obtain gene expression data and clinical information. The WGCNA analysis process of this step was performed in R 4.1.2 software (https://www.r‐project.org/); the goodSamplesGenes function was used to remove outliers and screen out the genes with the top 10% variance as input genes for analysis; clinical features (control samples and tumour samples) were imported; sample clustering was performed; pickSoftThreshold function was used to calculate soft threshold power; then, the adjacency was converted into a topological overlap matrix; hierarchical clustering and dynamic shearing was used; the minimum number of genes in the module was set to 30; and similar modules were merged; the gene modules related to clinical features of GC were constructed, and GC clinical module genes were identified.

Using ‘stomach cancer’ as the keyword, we searched the GeneCards database to obtain GC‐related targets, calculated the median of the Relevance score and screened out the targets that were greater than or equal to the median with a higher correlation with GC for subsequent analysis. Tbtools software^36^ was used to construct the Venn diagram of GC clinical module genes, GC database genes and EA related targets obtained by WGCNA, and the cross targets of EA and GC were obtained.

### 2.3 Construction of protein–protein interaction network of intersection targets and identification core candidate targets

We imported the intersection targets of EA/GC into the STRING 11.5 database^37^ (https://cn.string‐db.org/) to construct a protein–protein interaction (PPI) network and selected the creature as ‘*Homo sapiens*’; the confidence parameter was set as ‘medium confidence > 0.4’, exported the PPI network, used^37^ Cytoscape 3.8.2 software to visualize the network and used the cytoHubba plug‐in that comes with the software to screen core targets; this plugin is a powerful tool to help screen core targets; we choose four commonly used algorithms: Degree, MCC, Betweenness, Closeness. The TOP20 targets ranked by the score of each algorithm were extracted and mapped to the Venn diagram, and the intersection was determined as the core candidate target to improve the accuracy of screening.

### 2.4 Validation of core candidate targets with receiver operating characteristic curve and Kaplan–Meier survival analysis

We extracted the expression data of core candidate targets in the TCGA‐STAD dataset and used the bioinformatics online platform to generate the receiver operating characteristic (ROC) curve to verify the core candidate targets. The overall survival (OS) analysis of the core targets was performed using GC data from the Kaplan–Meier (KM) plotter (http://kmplot.com) to analyse the prognostic value of the core targets. The patients were divided into high expression group and low expression group according to the median expression level of mRNA, and the two groups were compared, and the hazard ratio with 95% confidence intervals and the log rank *p* value were calculated.

### 2.5 Gene Ontology functions of key targets and enrichment analysis of Kyoto Encyclopedia of Genes and Genomes pathways

We used the Metascape database^38^ (https://metascape.org/gp/index.html) for Gene Ontology (GO) function and Kyoto Encyclopedia of Genes and Genomes (KEGG) pathway enrichment analysis for key targets; the enrichment conditions were *p* < 0.01 and the minimum number of enriched genes ≥3, used KEGG Mapper to draw the KEGG map of the key signalling pathways that were significantly enriched, used bioinformatics online platform to visualize the top 15 biological processes (BP), cellular components (CC), molecular functions (MF) and the top 20 KEGG pathway enrichment results, used Cytoscape 3.8.2 software to build interaction network of EA, the key Targets, and the top 15 GO functional items (BP, CC and MF) and the top 20 KEGG pathways.

### 2.6 Molecular docking

We obtained the SDF format file of the ligand EA from the Pubchem database, used the Chem3D module of the ChemOffice 2018 software to minimize the energy of the EA and saved it in mol2 format, obtained the receptor protein structures of key targets from the RCSB PDB database (https://www.rcsb.org/),^39^ imported them into Pymol software for modification, removed water and other small molecules and used Autodock Tools v.1.5.6 software to process receptor proteins and ligands, converted to pdbqt formats and used Autodock Vina software^40^ to search for the best binding site to complete the molecular docking of receptor protein and ligand. After completion, use Pymol software to visualize the results. Single‐gene gene set enrichment analysis (GSEA) of key targets was performed using GSEA software.^41^ According to the expression data of TCGA‐STAD dataset, STAD samples were divided into high expression group and low expression group, and the background gene set was set to h.all.v2023.1.Hs.symbols.gmt.

### 2.7 Molecular dynamics simulation

We choose a protein–ligand complex with high binding energy as an example and use Gromacs software version 5.1.2 to perform molecular dynamics simulation of the complex to simulate the binding stability of protein and ligand, processed proteins with the AMBER99SB force field to obtain topology files, and Acpype's GAFF position to obtain topology files for ligands. The complexes were constructed; the protein–ligand complexes were defined in the unit box and filled with solvent water molecules for solvation; and then, ions were added to the system to keep the system charge neutral. Then, the systems were heated from 0 to 300 K, and the energy was minimized using the steepest descent method with 50,000 steps. The energy‐minimized systems were equilibrated with NVT and NPT with a step size of 2 fs for a total time of 100 ps by positional constraints, and the systems were brought into equilibrium at the set temperature and pressure, and then unconstrained for 10 ns MDS. After completing the MDS, the trajectories were analysed, and the gmx_rms and gmx_hbond programs in the Gromacs software package were introduced to calculate the root mean square deviation (RMSD) and H bonds between proteins, between backbones and between small molecule ligands; combined with APBS software version 3.0.0, the binding free energy of the protein–ligand complexes was calculated using the molecular mechanics Poisson–Boltzmann surface area (MM‐PBSA) method^41^, ^42^; the results were visualized with qtgrace software and Excel tables to assess the stability of the complexes MDS.

## 3 RESULTS

### 3.1 The potential targets for EA

**FIGURE 2:**
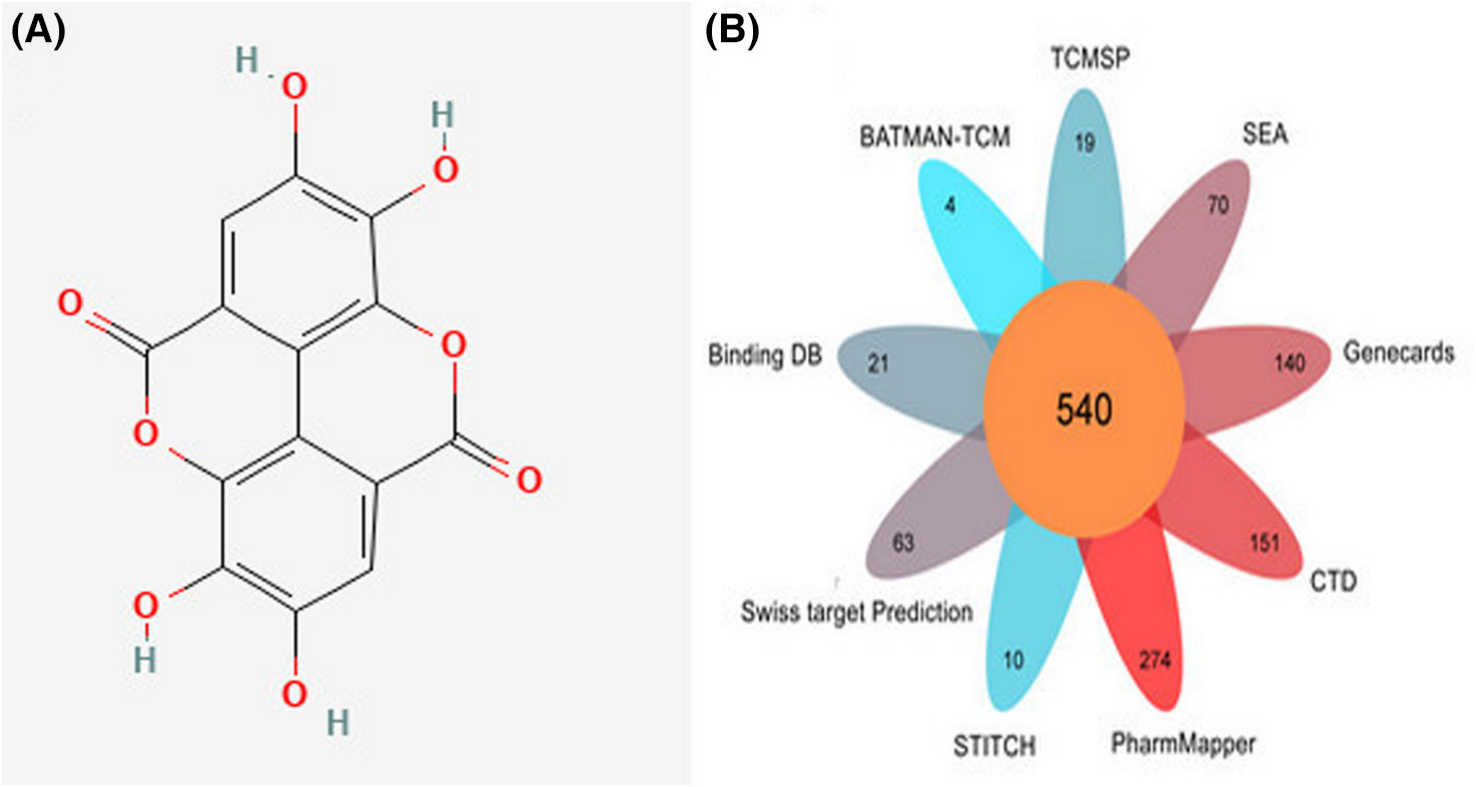
The structure of EA and acquisition of potential targets of EA. (A) Two‐dimensional structure of EA. (B) The number of EA candidate targets obtained from nine databases. EA, ellagic acid.

We obtained ADME parameters of EA from the TCMSP database, OB = 43.06% and DL = 0.43, where OB represents the speed and degree of drug absorption into human circulation, which is an important indicator for evaluating drug efficacy, and DL reflects the properties of drugs with specific functional groups or the same or similar physical properties,^43^ according to the recommendation of TCMSP database, OB ≥ 30% and DL ≥ 0.18 are commonly used criteria for screening effective active ingredients. Based on these two powerful parameters, we infer that EA can exert effective pharmacological activity in the body, and the structure of EA was shown in Figure 2A.

We collected candidate targets of EA from nine databases, namely TCMSP, BATMAN‐TCM, Binding DB, SwissTargetPrediction, STITCH, pharmmapper, CTD, GeneCards and SEA database, as shown in Figure 2B; the acquired targets were standardized and named through the UniProt database; and after merging and deduplication, 540 EA candidate targets were finally obtained.

### 3.2 The disease targets for GC and intersection targets for EA against GC

**FIGURE 3:**
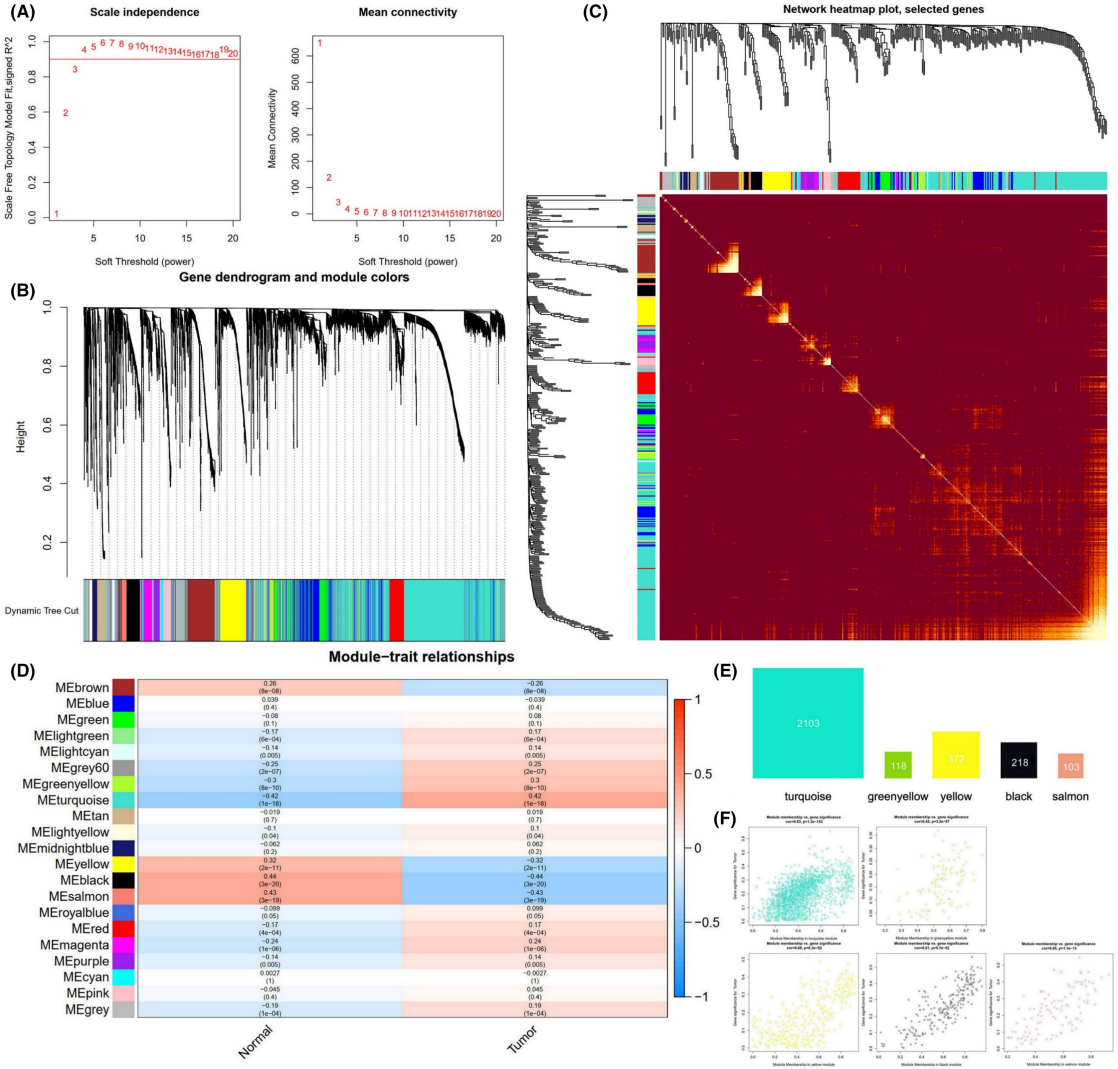
Construction of weighted co‐expression gene network and obtain the GC clinical module genes. (A) The soft threshold calculation. (B) The co‐expression network clustering dendrogram. (C) The heat map of top 400 gene. (D) Module and clinical feature similarity heat map. (E) The number of genes contained in five modules of turquoise, green‐yellow, black, salmon and yellow. (F) Scatter plots of the correlation between genes and tumour tissue in five modules. GC, gastric cancer.

We obtained data on TCGA‐STAD patients from the TCGA database, which included 32 control samples and 375 tumour samples. A total of 57,067 genes were obtained by removing outliers, and 5707 genes with the top 10% variance were screened for analysis. The WGCNA package was used to construct a weighted gene co‐expression network, selecting a soft threshold *β* = 4 was set, and a scale‐free network was constructed with *R* ^2^ ≈ 0.95, as shown in Figure 3A; 21 modules was constructed, as shown in Figure 3B; the heat map of the top 400 genes was visualized, as shown in Figure 3C; clinical features were loaded, that is control samples and tumour samples, and the correlation between modules and clinical features was calculated, as shown Figure 3D; Among them, the turquoise module and the green‐yellow module had a strong positive correlation with GC tissues, containing 2103 and 118 genes, respectively; the black module, salmon module and yellow module had strong negative correlations, containing 218, 103 and 372 genes, respectively. We included the genes contained in these five modules into this study, and finally, 2914 GC clinical module genes were obtained for subsequent analysis, the number of genes included in the five modules and the scatter plots of the included genes and tumour tissue correlations were shown in Figure 3E,F.

To make the obtained GC‐related genes more accurate, we obtain the intersection of the targets of GeneCards database and GC clinical module genes of WGCNA as GC‐related targets for subsequent analysis. We used ‘Gastric cancer’ as keyword to search the GeneCards database and obtained 5475 GC‐related targets. With the relevance score greater than or equal to the median 0.784, 2792 targets with higher correlation with the disease were selected for subsequent analysis.

**FIGURE 4:**
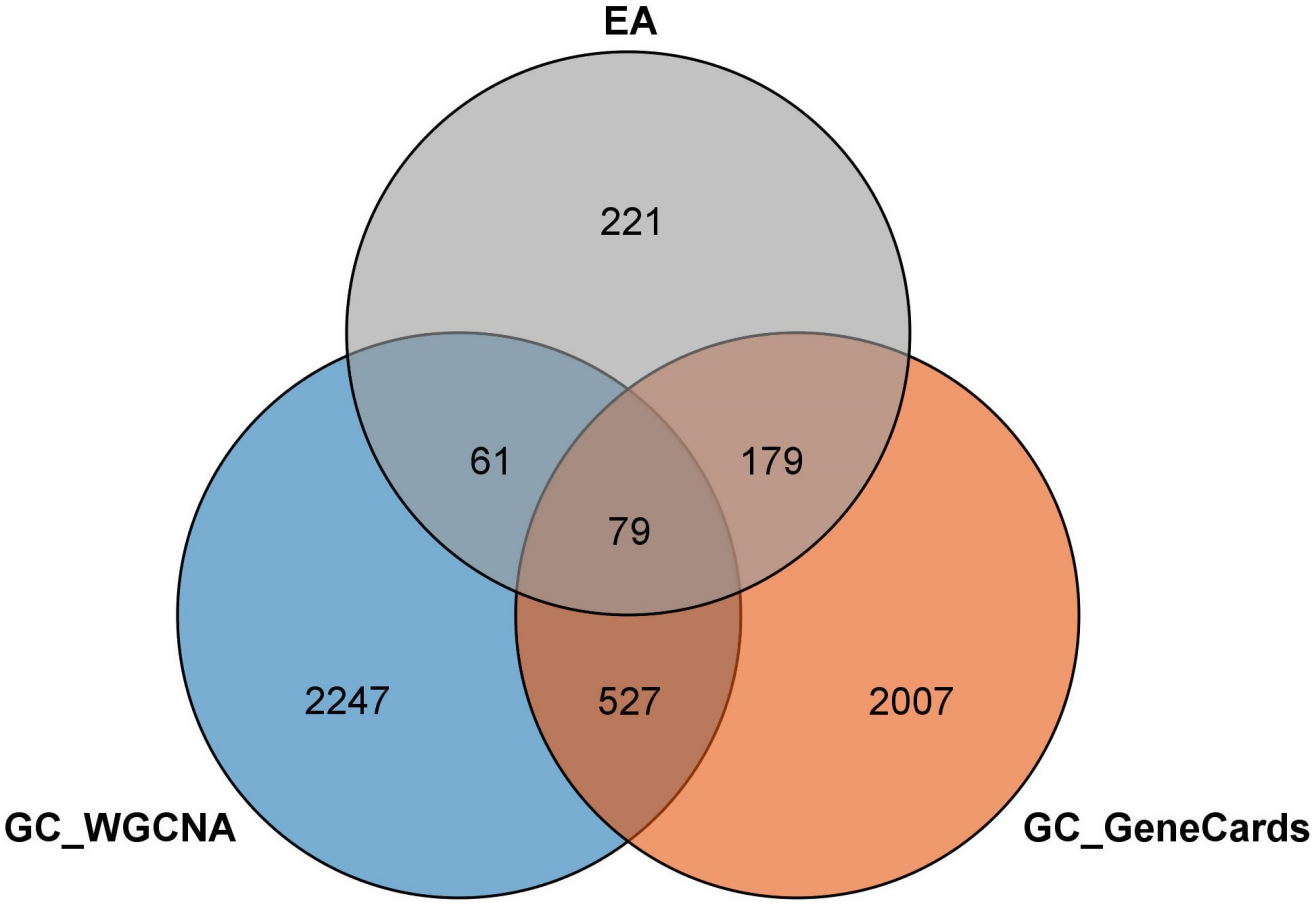
Venn diagram of GC clinical module genes, GC database related genes and EA related candidate targets. EA, ellagic acid; GC, gastric cancer.

Tbtools software was used to construct the Venn diagram to obtain the intersection of EA and GC. The intersection Venn diagram contains three parts, which are GC clinical module genes, GC database related genes, and EA related candidate targets. By constructing the Venn diagram, 79 intersection targets of EA and GC were obtained, as shown in Figure 4.

### 3.3 Construction of PPI network of intersection targets and identification core candidate targets

**FIGURE 5:**
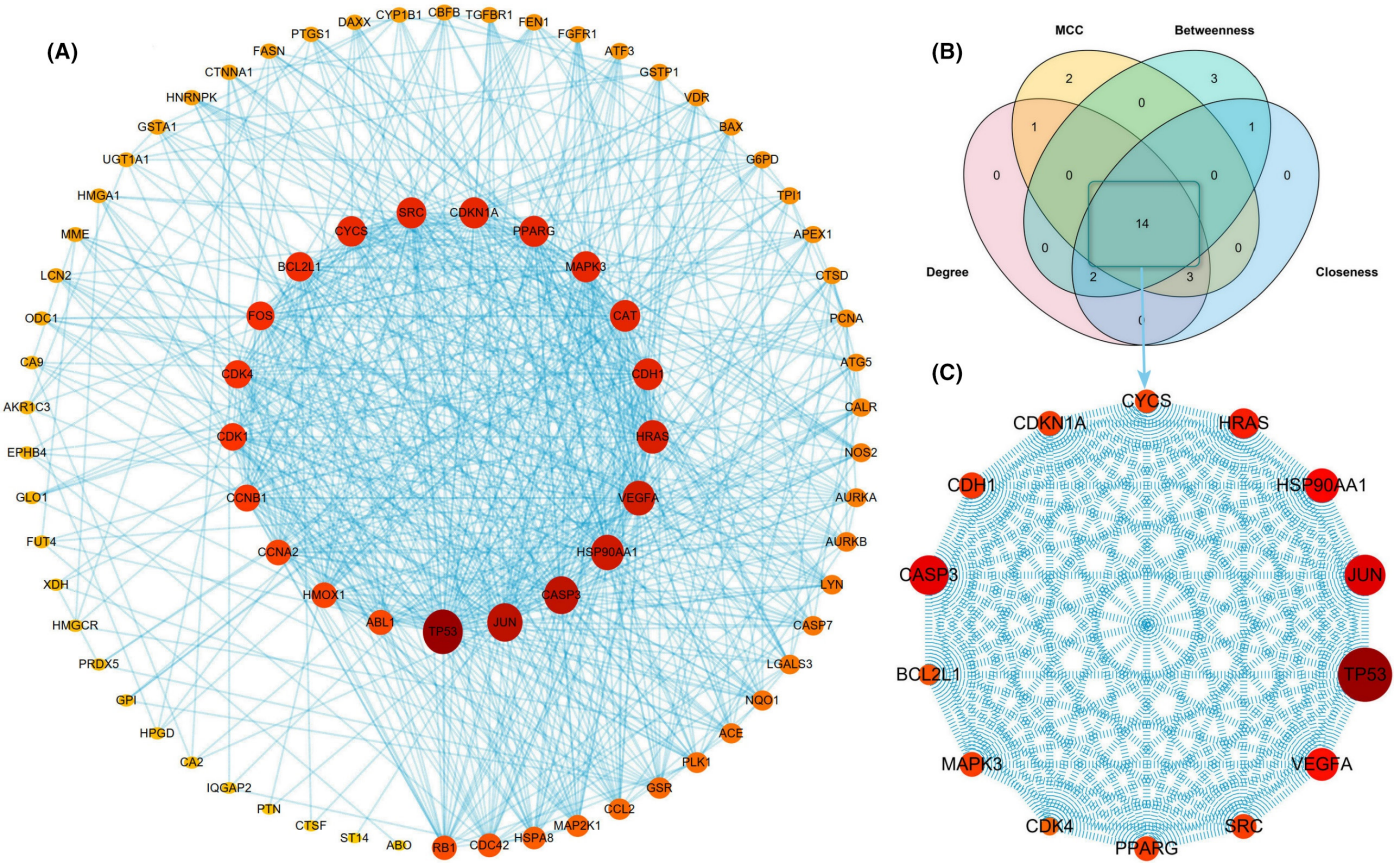
Construction of PPI network and screening of core candidate targets. (A) PPI network of EA/GC cross targets, the colour and area of the target are proportional to the Degree value. (B) Degree, MCC, Betweenness, Closeness algorithm Top 20 Venn diagram of target intersection. (C) The interaction network of core candidate targets. EA, ellagic acid; GC, gastric cancer; PPI, protein–protein interaction.

**FIGURE 6:**
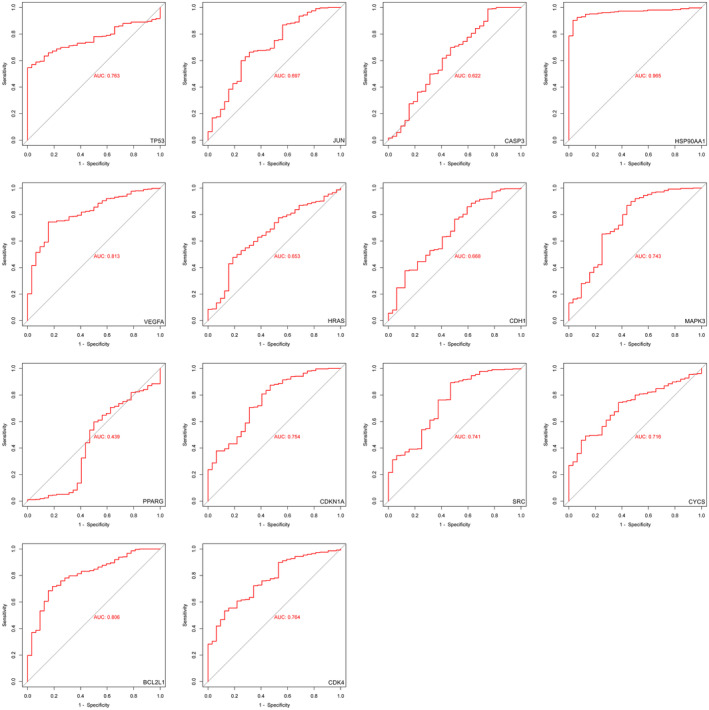
ROC curve validation of TP53, JUN, CASP3, HSP90AA1, VEGFA, HRAS, CDH1, MAPK3, PPARG, CDKN1A, SRC, CYCS, BCL2L1 and CDK4. ROC, receiver operating characteristic.

We imported the obtained 79 intersection targets of EA/GC into the STRING 11.5 database to construct a PPI network, selected the organism as ‘*Homo sapiens*’ and set the confidence parameter as ‘medium confidence > 0.4’. A PPI network with 79 nodes and 678 edges was obtained, which was visualized with Cytoscape 3.8.2 software, as shown in Figure 5A, and then, the Degree, MCC, Betweenness and Closeness algorithms of the software's cytoHubba plugin were used to screen core candidate targets; the Top 20 targets ranked by the scores of the four algorithms were extracted and mapped to the Venn diagram; and the intersection was determined as the core candidate target, as shown in Figure 5B. A total of 14 core candidate targets were obtained, as shown in Figure 6C, which are TP53, JUN, CASP3, HSP90AA1, VEGFA, HRAS, CDH1, MAPK3, PPARG, CDKN1A, SRC, CYCS, BCL2L1 and CDK4; the parameters of the core candidate targets were shown in Table 1.

**TABLE 1:** Parameters of core candidate targets.

Gene name	Protein name	Degree	MCC	Betweenness	Closeness
TP53	Cellular tumour antigen p53	57	2.26027E+13	785.1792282	67.5
JUN	Transcription factor Jun	47	2.26026E+13	355.7607835	62.33333333
CASP3	Caspase‐3	46	2.26027E+13	281.5796564	61.83333333
HSP90AA1	Heat shock protein HSP 90‐alpha	42	2.26022E+13	227.1011829	59.83333333
VEGFA	Vascular endothelial growth factor A	41	2.25009E+13	398.2994267	59.5
HRAS	GTPase HRas	39	2.2601E+13	265.1134173	58.33333333
CDH1	Cadherin‐1	36	2.2506E+13	460.0906835	56.5
MAPK3	Mitogen‐activated protein kinase 3	35	1.40414E+12	119.8652477	56.33333333
PPARG	Peroxisome proliferator‐activated receptor gamma	35	1.774E+11	409.9211882	56.33333333
CDKN1A	Cyclin‐dependent kinase inhibitor 1	35	2.25943E+13	109.269121	55.83333333
SRC	Proto‐oncogene tyrosine‐protein kinase Src	35	2.2602E+13	181.9932824	56.33333333
CYCS	Cytochrome c	34	2.24942E+13	115.110357	55.83333333
BCL2L1	Bcl‐2‐like protein 1	32	2.25957E+13	166.485164	54.83333333
CDK4	Cyclin‐dependent kinase 4	30	2.11986E+13	63.38586588	53.5

### 3.4 Validation of core candidate targets with ROC curve and Kaplan–Meier survival analysis

We extracted the expression data of the core candidate targets in the TCGA‐STAD dataset and generated the ROC curve to verify the core candidate targets. When the area under the curve (AUC) was within the range of (0.5, 1), it indicated that the gene had good predictive ability. According to the ROC curves of the core candidate targets, we generated, as shown in Figure 6, the results showed that among the 14 core candidate targets, except for PPARG, the AUC values of TP53, JUN, CASP3, HSP90AA1, VEGFA, HRAS, CDH1, MAPK3, CDKN1A, SRC, CYCS, BCL2L1 and CDK4 were all at (0.5, 1) range, indicating that these 13 key targets are closely related to GC.

**FIGURE 7:**
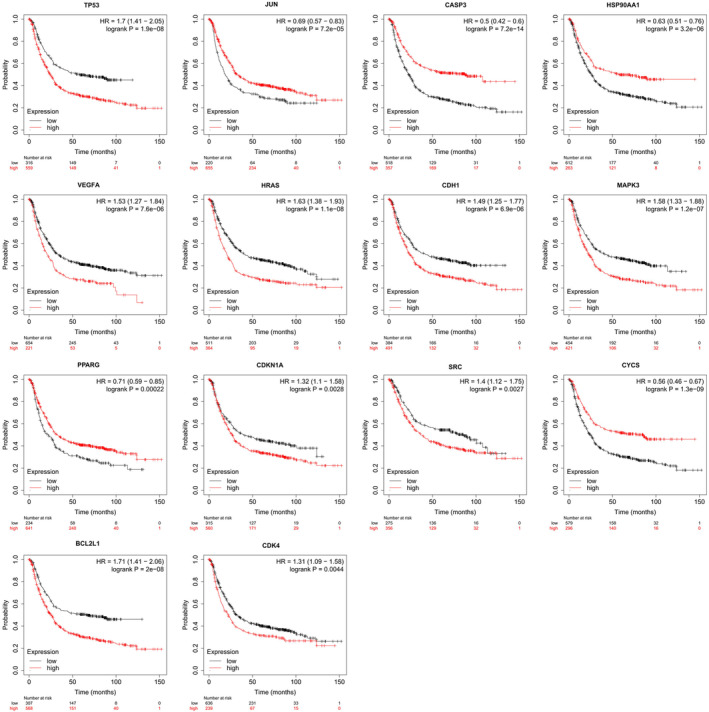
The KM survival analysis of TP53, JUN, CASP3, HSP90AA1, VEGFA, HRAS, CDH1, MAPK3, PPARG, CDKN1A, SRC, CYCS, BCL2L1 and CDK4. KM, Kaplan–Meier.

Then, in order to further reveal the prognostic value of 14 core targets, Kaplan–Meier plotter was used to analyse the survival of core targets, as shown in Figure 7. The results showed that 14 core targets TP53, JUN, CASP3, HSP90AA1, VEGFA, HRAS, CDH1, MAPK3, PPARG, CDKN1A, SRC, CYCS, BCL2L1 and CDK4 were significantly associated with poor prognosis in GC (*p* < 0.05). Based on the results of ROC curve and KM survival analysis, we predicted that 13 key targets TP53, JUN, CASP3, HSP90AA1, VEGFA, HRAS, CDH1, MAPK3, CDKN1A, SRC, CYCS, BCL2L1 and CDK4 may be the key targets of EA against GC.

### 3.5 Enrichment analysis of GO and KEGG pathway

**FIGURE 8:**
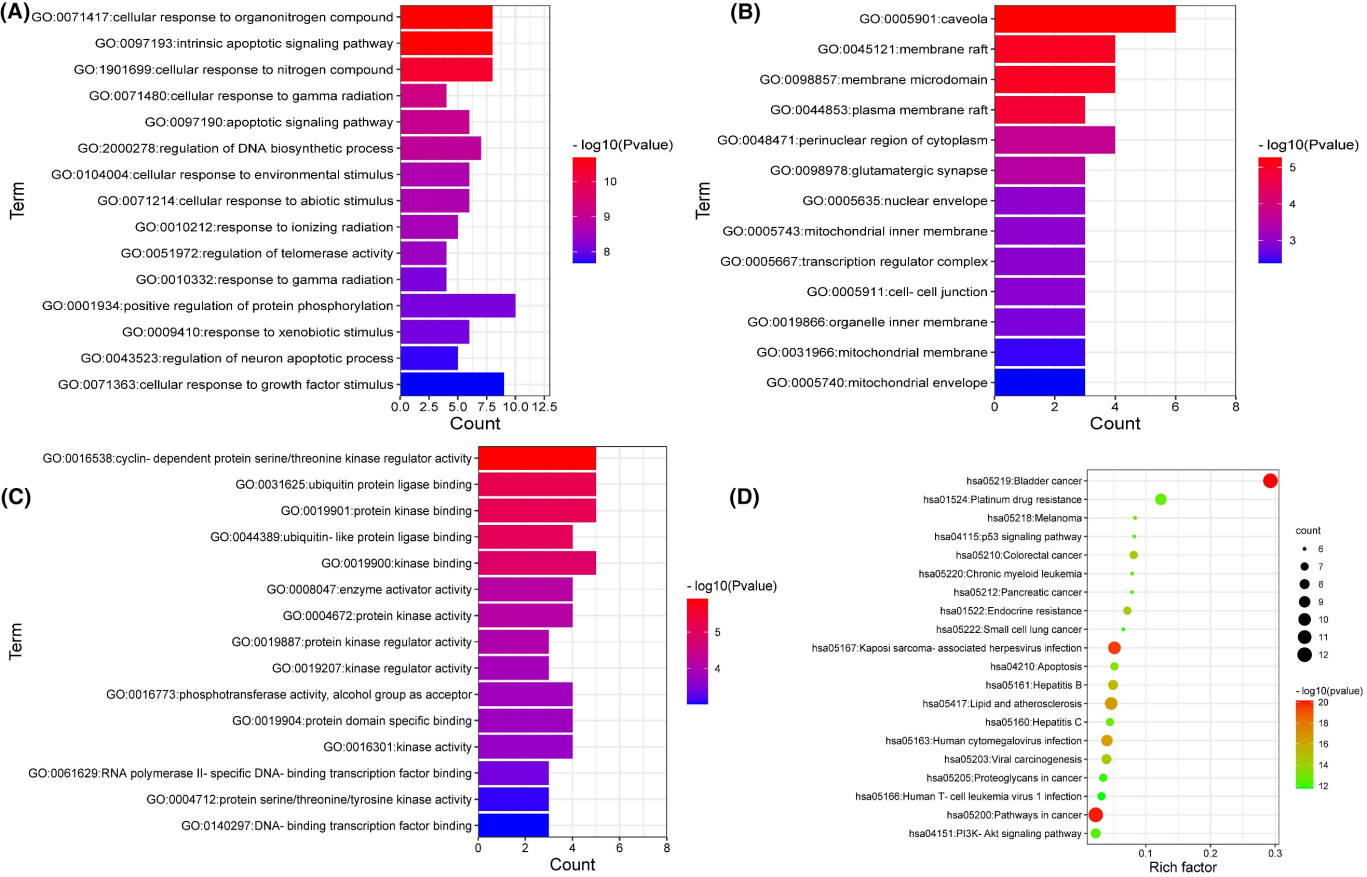
GO function and KEGG pathway enrichment analysis of key targets. (A) The top 15 GO items in BP. (B) The 13 GO items in CC. (C) The top 15 GO items in MF. (D) The top 20 KEGG pathways. BP, biological processes; CC, cellular components; GO, Gene Ontology; KEGG, Kyoto Encyclopedia of Genes and Genomes; MF, molecular functions.

We used the Metascape database to perform enrichment analysis of GO functions and KEGG pathways for 13 key targets; under the conditions of *p* < 0.01 and the minimum number of enriched genes ≥3, a total of 283 GO entries and 99 KEGG pathways were obtained. The results with significant enrichment were visualized in order of *p* value, as shown in Figure 8. Among them, there were 253 BPs, which were mainly enriched in the cellular response to organonitrogen compound, intrinsic apoptotic signalling pathway, cellular response to nitrogen compound, cellular response to gamma radiation, apoptotic signalling pathway, regulation of DNA biosynthetic process, cellular response to abiotic stimulus and cellular response to an environmental stimulus; there were 13 CCs, which were mainly enriched in caveola, membrane raft, membrane microdomain, plasma membrane raft, perinuclear region of cytoplasm, glutamatergic synapse, nuclear envelope and mitochondrial inner membrane; there were 17 MFs, which were mainly enriched in cyclin‐dependent protein serine/threonine kinase regulator activity, ubiquitin protein ligase binding, protein kinase binding, ubiquitin‐like protein ligase binding, kinase binding, enzyme activator activity, protein kinase activity and protein kinase regulator activity.

**FIGURE 9:**
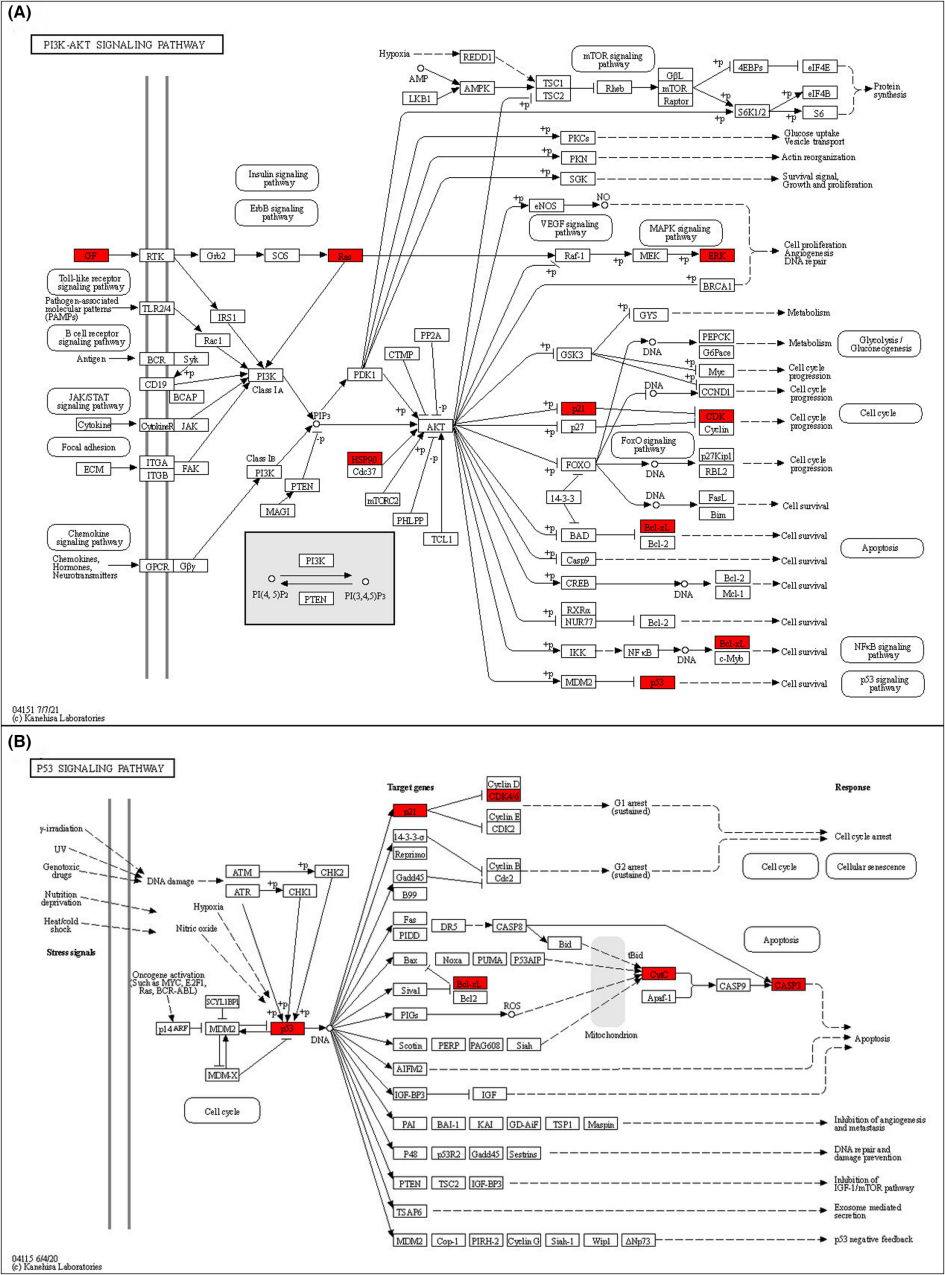
KEGG maps of the key target distribution. (A) The PI3K‐Akt signalling pathway. (B) The p53 signalling pathway. The white represents other targets of the pathway, and red represents the key target of EA against GC. EA, ellagic acid; GC, gastric cancer; KEGG, Kyoto Encyclopedia of Genes and Genomes.

Among the top 20 KEGG pathways from KEGG pathway enrichment results, signalling pathways related to GC development included pathways in cancer, viral infection, lipid and atherosclerosis, endocrine resistance, apoptosis, platinum drug resistance, p53 signalling pathway, other cancer‐related pathways, etc. KEGG Mapper was used to draw KEGG maps of the distribution of key targets in the p53 signalling pathway and PI3K‐Akt signalling pathway, as shown in Figure 9. The red colour in the figure represented the key targets.

**FIGURE 10:**
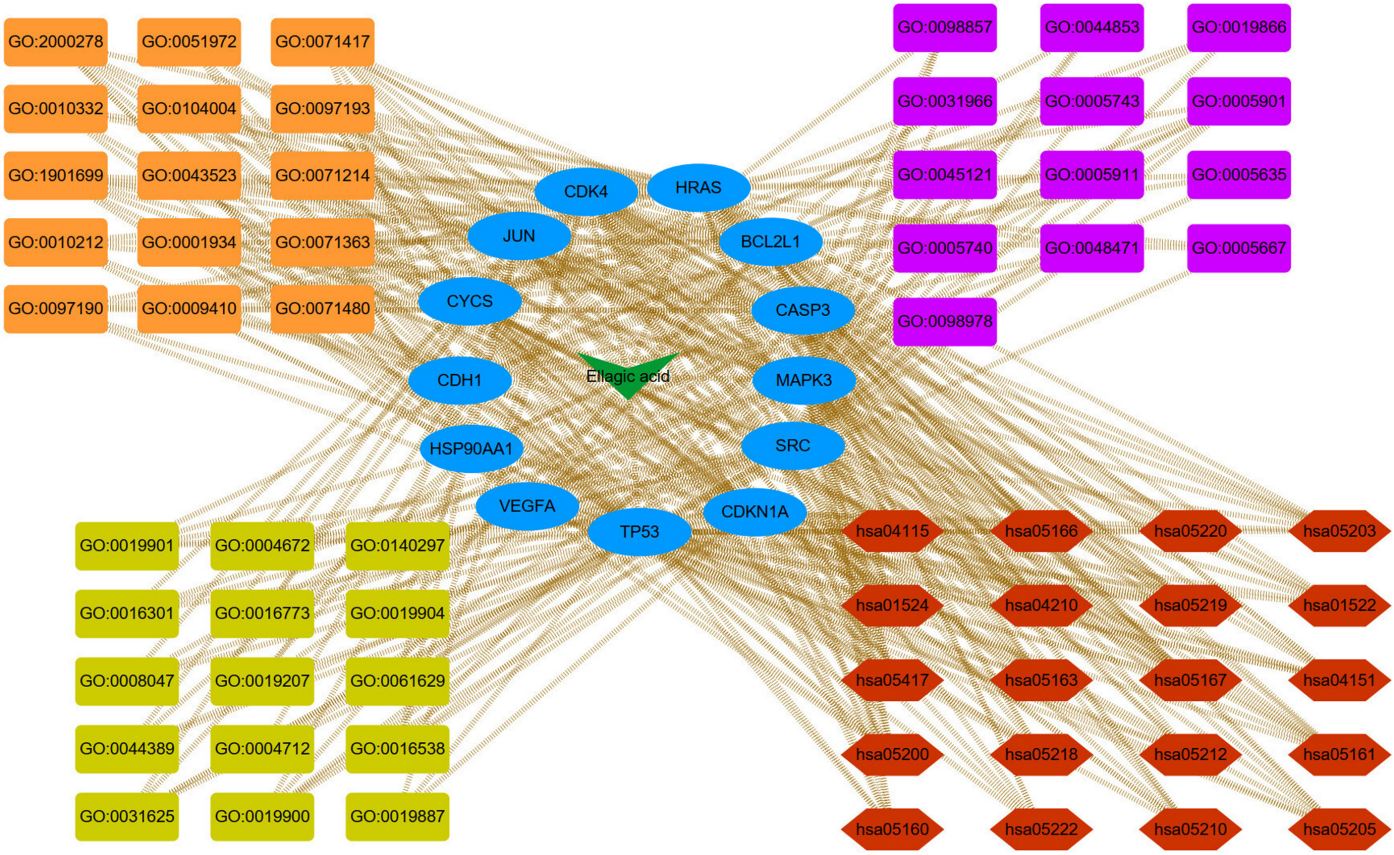
The interaction network of EA related targets‐13 key targets‐top 15 GO entries (BP, CC and MF)‐top 20 KEGG pathways. The green diamonds represent EA, blue circles represent 13 key targets, and orange rectangles represent top 15 BP entries, purple rectangles represent 13 CC entries, and red hexagons represent top 20 KEGG pathways. BP, biological processes; CC, cellular components; EA, ellagic acid; GO, Gene Ontology; KEGG, Kyoto Encyclopedia of Genes and Genomes; MF, molecular functions.

Then, Cytoscape 3.8.2 software was used to construct the interaction network of EA related targets‐key targets‐top 15 GO entries (BP, CC and MF)‐top 20 KEGG pathways, as shown in Figure 10.

### 3.6 Molecular docking

**FIGURE 11:**
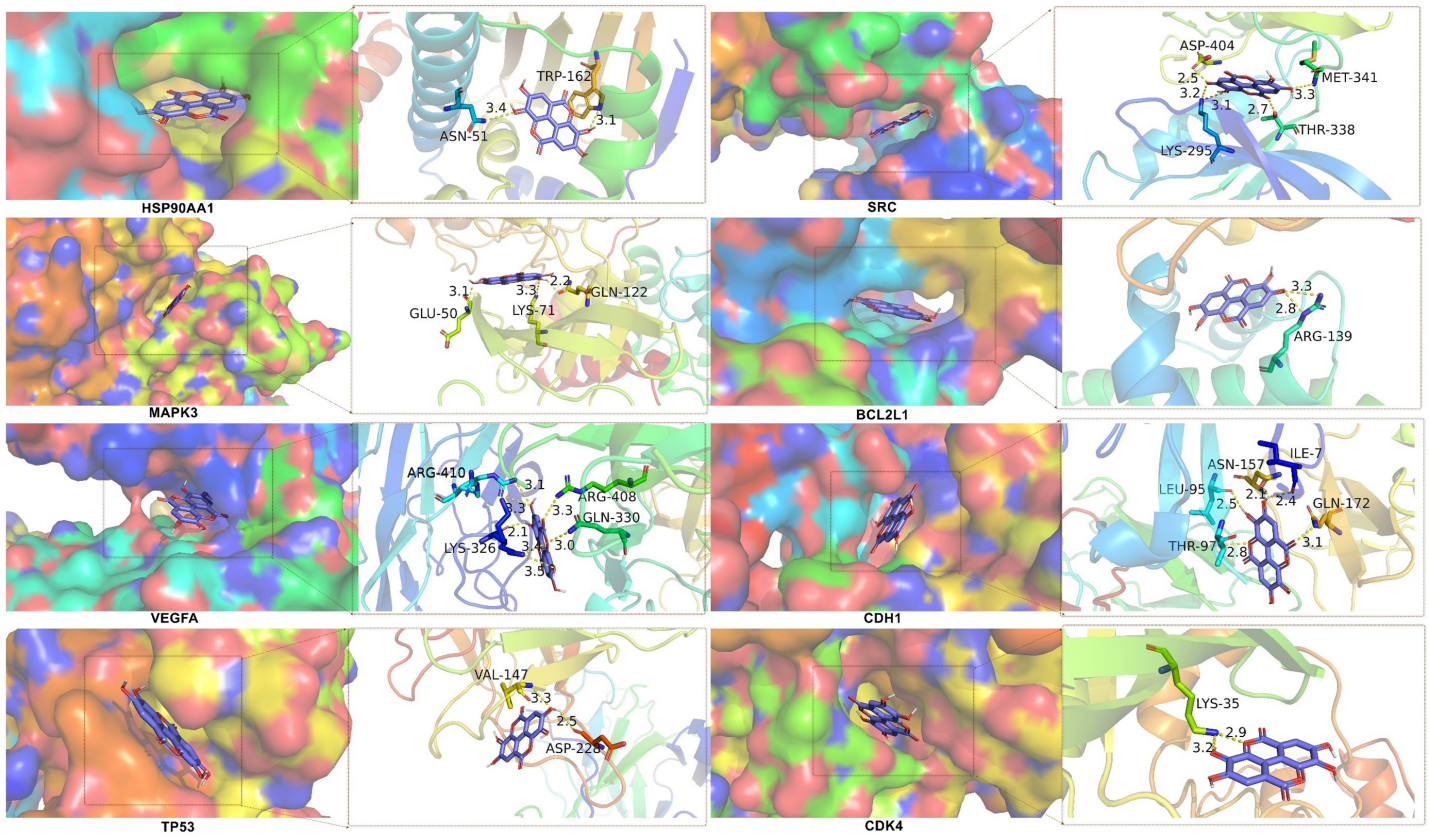
Molecular docking modes ranked top 8 in terms of binding energy between EA and key targets. EA, ellagic acid.

**FIGURE 12:**
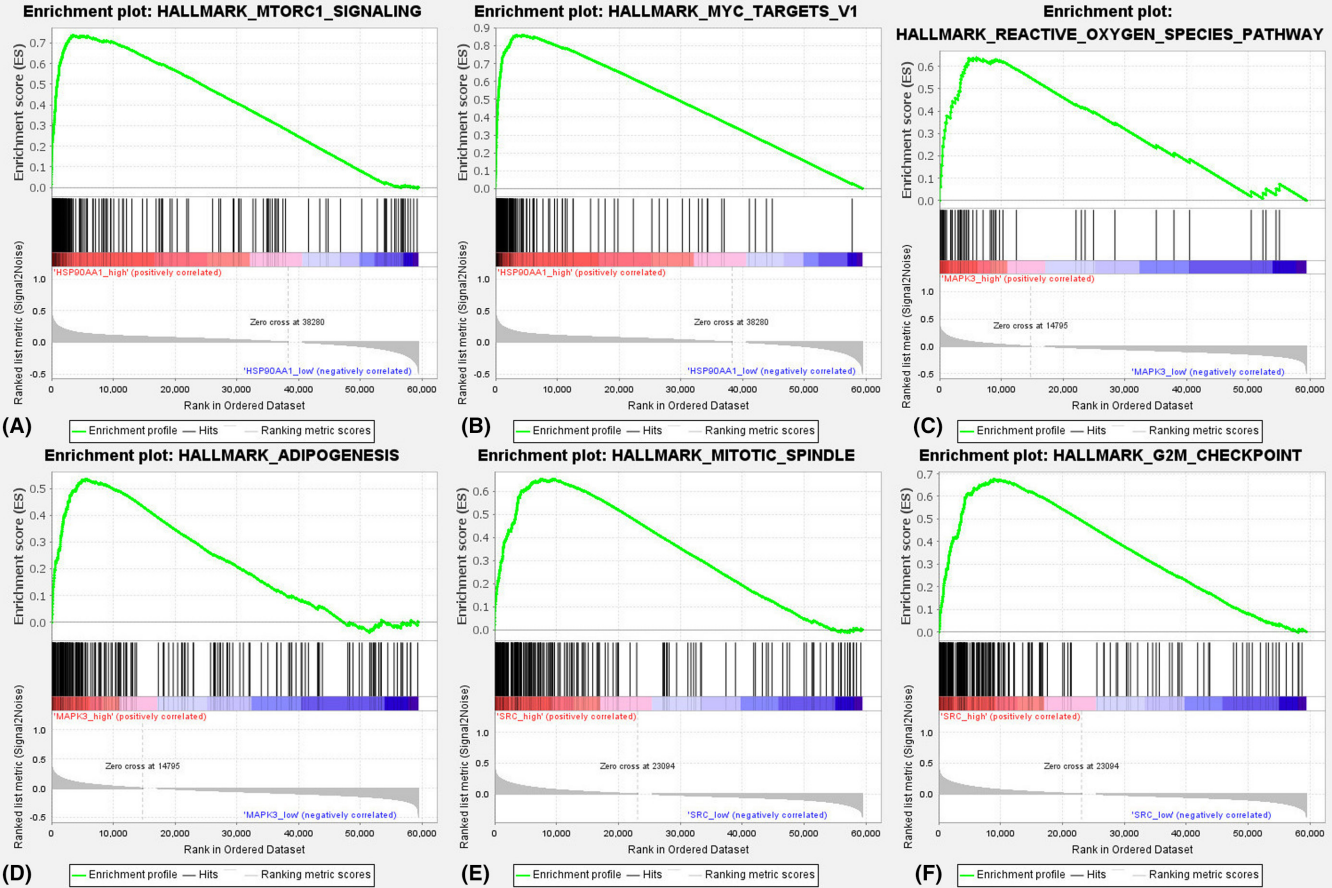
GSEA analysis. (A) GSEA analysis of HSP90AA1 in HALLMARK_MTORC1_SIGNALING; (B) GSEA analysis of HSP90AA1 in HALLMARK_MYC_TARGETS_V1; (C) GSEA analysis of MAPK3 in HALLMARK_REACTIVE_OXYGEN_SPECIES_PATHWAY; (D) GSEA analysis of MAPK3 in HALLMARK_ADIPOGENESIS; (E) GSEA analysis of SRC in lysosome HALLMARK_MITOTIC_SPINDLE; (F) GSEA analysis of SRC in lysosome HALLMARK_G2M_CHECKPOINT. GSEA, gene set enrichment analysis.

We used Autodock Tools v.1.5.6 and Autodock Vina software to perform molecular docking of EA with key target receptor proteins to evaluate the interaction of EA with key targets and verify the accuracy of the above analysis. It is generally believed that the binding energy less than 0 kcal/mol indicates the existence of binding activity between protein ligands, and the binding energy less than −5.0 kcal/mol indicates the existence of good binding activity.^44^ The binding energy of docking between EA and 13 key target receptor protein molecules, the number of H‐bonds formed, the amino acid residues involved in the H‐bond and the bond length were shown in Table 2. According to the results of our molecular docking, the molecular docking of EA with key target receptor proteins was plotted with the top 8 binding energy as an example, as shown in Figure 11. The binding energies of EA to 13 key targets TP53, JUN, CASP3, HSP90AA1, VEGFA, HRAS, CDH1, MAPK3, CDKN1A, SRC, CYCS, BCL2L1 and CDK4 were all less than −5.0 kcal/mol, indicating that EA can effectively bind to the receptor proteins of key targets, revealing that these key targets may play an important role in EA anti‐GC. Among them, HSP90AA1‐EA, MAPK3‐EA and SRC‐EA have the highest binding energy, which may be valuable key targets. Therefore, we performed a single‐gene GSEA analysis of these three targets and found that they were mainly enriched in the HALLMARK_MTORC1_SIGNALING, HALLMARK_MYC_TARGETS_V1, HALLMARK_REACTIVE_OXYGEN_SPECIES_PATHWAY, HALLMARK_ADIPOGENESIS, HALLMARK_MITOTIC_SPINDLE and HALLMARK_G2M_CHECKPOINT, respectively, as shown in Figure 12.

**TABLE 2:** Binding energies of EA docking with 13 key target receptor protein molecules, the number of H‐bonds formed, the amino acid residues involved in the H‐bond and the bond length.

Uniprot_ID	Gene name	PDB_ID	Binding energy (kcal/mol)	No. of H‐Bonds	Amino acid residues involved in H‐bonding & bond length (Å)
P04637	TP53	3ZME	−8.3	2	Val147‐O(3.3); Asp228‐O(2.5)
P05412	JUN	6Y3V	−7.1	11	Arg60‐O(3.1); Arg60‐O(3.0); Arg56‐O(3.3); Arg56‐O(3.2); Arg56‐O(3.3); Arg129‐O(3.0); Arg129‐O(3.1); Asn175‐O(3.2); Asn225‐O(3.3); Asn225‐O(3.4); Asn225‐H(2.1)
P42574	CASP3	2XYG	−6.8	7	Phe250‐O(3.3); Phe250‐O(3.4); Ser209‐O(3.1); Ser209‐O(3.4); Ser209‐O(3.0); Arg207‐O(3.1); Tyr204‐O(3.1)
P07900	HSP90AA1	2YK9	−10.6	2	Asn51‐O(3.4); Trp162‐O(3.1)
P15692	VEGFA	5DN2	−8.5	7	Arg410‐O(3.3); Arg410‐O(3.1); Arg408‐O(3.3); Lys326‐H(2.1); Lys326‐O(3.4); Lys326‐O(3.5); Gln330‐O(3.0)
P01112	HRAS	2CE2	−8.1	5	Ala18‐O(3.0);Ser17‐0(3.0);Ser17‐0(3.1);Ser17‐0(3.0);Gly60‐O(3.1)
P12830	CDH1	3FF7	−8.4	5	Leu95‐H(2.5); Thr97‐O(2.8); Asn157‐H(2.1); Ile7‐H(2.4); Gln172‐O(3.1)
P27361	MAPK3	6GES	−9	3	Glu50‐O(3.1); Lys71‐O(3.3); Gln122‐H(2.2)
P38936	CDKN1A	6P8H	−7.7	4	Asn221‐O(3.5); Asn221‐O(3.0); Asn222‐O(3.2); Asn222‐O(3.3)
P12931	SRC	2BDJ	−9.5	5	Asp404‐O(2.5); Lys295‐O(3.2); Lys295‐O(3.1); Thr338‐0(2.7); Met341‐O(3.3)
P99999	CYCS	3ZCF	−7.8	2	His18‐O(3.1); Asn54‐H(2.0)
Q07817	BCL2L1	3ZK6	−8.8	2	Arg139‐O(2.8); Arg139‐O(3.3)
P11802	CDK4	2W9Z	−8.2	2	Lys35‐O(3.2); Lys35‐O(2.9)

### 3.7 Molecular dynamics simulation

**FIGURE 13:**
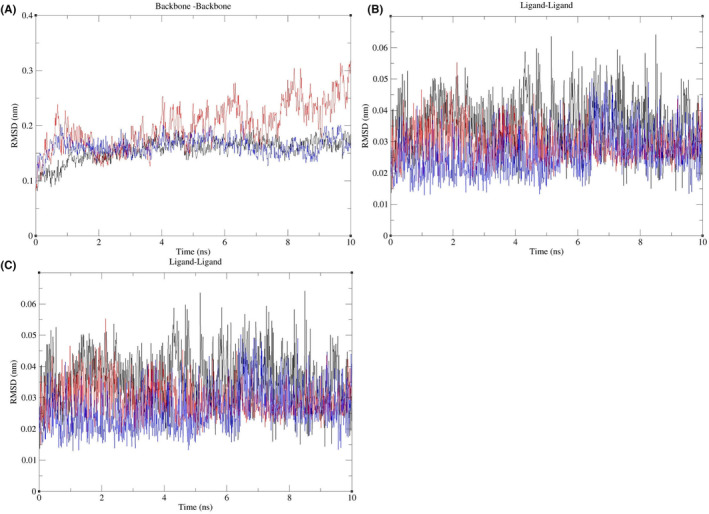
RMSD analysis of molecular dynamics simulations. (A) The RMSD of protein backbone‐protein backbone. (B) The RMSD of ligand–ligand. (C) The RMSD of protein–protein. The black represents the complex of EA and HSP90AA1, red represents the complex of EA and MAPK3, and blue represents the complex of EA and SRC. EA, ellagic acid; RMSD, root mean square deviation.

We selected the protein–ligand complexes of EA with HSP90AA1, EA with MAPK3 and EA with SRC as examples for molecular dynamics simulation to evaluate the binding stability and binding ability of proteins and ligands. The RMSD and H were calculated to evaluate the binding stability of protein–ligand complexes; as shown in Figure 13, we calculated the RMSD of backbone‐backbone, ligand–ligand and protein–protein. In terms of RMSD of backbone‐backbone, the mean ± standard deviation (SD) of EA and HSP90AA1 was 0.156 ± 0.021 (nm), the mean ± SD of MAPK3 was 0.033 ± 0.041 (nm), the mean ± SD of SRC was 0.163 ± 0.016 (nm); in terms of RMSD of ligand–ligand, the mean ± SD of EA and HSP90AA1 was 0.036 ± 0.009 (nm), the mean ± SD of MAPK3 was 0.029 ± 0.005 (nm), the mean ± SD of SRC was 0.026 ± 0.007 (nm); in terms of RMSD of protein–protein, the mean ± SD of EA and HSP90AA1 was 0.218 ± 0.029 (nm), the mean ± SD of MAPK3 was 0.236 ± 0.040 (nm), the mean ± SD of SRC was 0.216 ± 0.018 (nm). The results showed that EA formed stable protein–ligand complexes with HSP90AA1, MAPK3 and SRC, which all tended to equilibrium after 3 ns, and did not fluctuate significantly during the simulation.

**FIGURE 14:**
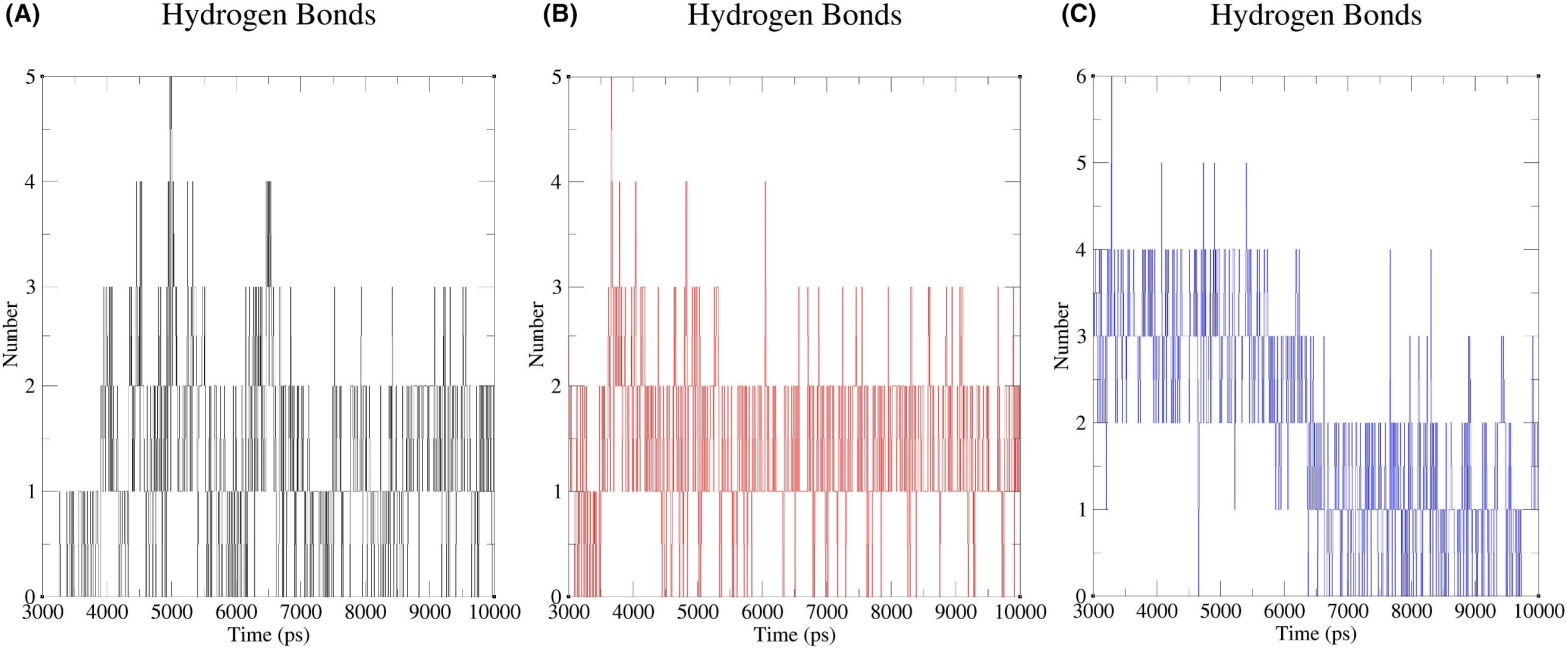
The time‐dependent analysis of hydrogen bonds between proteins and ligands in 3–10 ns MDS. (A) The complex of EA and HSP90AA1. (B) The complex of EA and MAPK3. (C) The complex of EA and SRC. EA, ellagic acid; MDS, molecular dynamics simulations.

The hydrogen‐bonding interaction has an important contribution to the stability of protein–ligand complexes. We analysed the 3–10 ns MDS trajectories of the stabilized systems for hydrogen‐bonding interaction analysis and calculated the number of hydrogen bonds formed between proteins and ligands in the protein–ligand complexes of EA and HSP90AA1, EA and MAPK3, and EA and SRC, as shown in Figure 14. The results showed that the number of H bonds of proteins and ligands in the complexes of EA and HSP90AA1, EA and MAPK3 were stable from 0 to 3 to the end, with a maximum of 5, when the number of H bonds of proteins and ligands in the complex of EA and SRC is 3–7 ns, the number of H bonds is mainly concentrated in 2–4, 7 ns to the end, and remains stable at 0–2 to the end. In conclusion, the number of H‐bonds formed by all protein–ligand complexes is stable during the simulation, and its dynamic changes may be increased or decreased due to the shift of atomic positions.

We performed MM‐PBSA binding energy calculations on the 3–10 ns MDS trajectories after stabilization, providing a more accurate estimate of binding affinity based on the binding free energy. We calculated van der Waals energy, electrostatic energy, polar solvation energy, nonpolar solvation energy and binding energy, as shown in Table 3, where the van der Waals energy, electrostatic energy and nonpolar solvation energy were all negative values, indicating that these interactions could promote the binding between the protein and the ligand, and had a large contribution to the binding energy, the polar solvation energy was positive and contributes little to ligand‐protein binding, the results of the binding energy of the three complexes showed that EA had a good binding force with the three target proteins, forming a stable complex, the binding energy of HSP90AA1 and EA is the highest, followed by MAPK3, and then SRC. The results of binding free energy seem to support the results of molecular docking.

**TABLE 3:** Calculation analysis of binding free energy of three protein–ligand complexes.

Component	ΔEvdW (kJ/mol)	ΔEelec (kJ/mol)	ΔGpolar (kJ/mol)	ΔGnonpolar (kJ/mol)	ΔGbind (kJ/mol)
HSP90AA1‐EA	−171.613	−24.497	132.248	−16.747	−64.011
MAPK3‐EA	−152.52	−47.292	154.37	−16.848	−40.165
SRC‐EA	−127.553	−28.762	109.183	−15.409	−39.497

Abbreviations: ΔEelec, electrostatic energy; ΔEvdW, van der Waals energy; ΔGbind, binding free energy; ΔGnonpolar, nonpolar solvation energy; ΔGpolar, polar solvation energy.

## 4 DISCUSSION

Currently, GC remains one of the most prevalent cancers worldwide, with high mortality rates, especially in developing countries. In recent years, more and more studies have shown that many natural components in plants can enhance sensitivity to chemotherapy and have synergistic effects with anti‐GC drugs,^45^, ^46^ and because of their good safety, they have broad development prospects in the prevention and treatment of GC. Most of the anti‐tumour active ingredients exert their anti‐tumour effects mainly by changing the proliferation, apoptosis, invasion and migration capabilities of cancer cells. According to previous literature studies and ADME parameters, EA may exert effective pharmacological activity in the body, EA is a natural phenolic compound that exists in a variety of fruits, and studies have shown that EA has anticancer effects,^9^, ^47^ the recent studies have shown that EA has anticancer properties on GC, but the related pharmacological mechanism needs further study.

Currently, network pharmacology has become a powerful tool for predicting the molecular mechanisms of drug–disease interactions; however, limitations such as lack of clinical information may limit the value and application of network pharmacology.^48^ In this study, bioinformatics analysis and the WGCNA method were introduced to identify gene modules closely related to the clinical characteristics of GC to compensate for the lack of clinical information in network pharmacology. Calculate the targets with higher GC correlation with the median Relevance score in the GeneCards database, take the intersection with the gene module targets for secondary screening, and use them as candidate targets for disease for subsequent analysis to improve the accuracy of GC‐related targets. Then, PPI network was constructed to obtain the candidate core targets of EA against GC, and the key targets were screened by KM survival analysis and ROC curve analysis. The enrichment analysis of key targets was performed to identify biological processes and molecular pathways related to the action of EA. With the development of artificial intelligence, computer‐aided drug design has become one of the key methods of contemporary preclinical drug discovery, combining computational techniques and related software programs to greatly improve the efficiency of helping to develop potential drugs,^49^ saving the cost of development. In the present study, molecular docking was used to calculate the interaction and binding force between EA and key targets. Then, the protein–ligand complex with high binding energy was selected as an example for MDS to evaluate the binding stability of the proteins and ligands, and the accuracy of key targets prediction was preliminarily verified.

Through network pharmacology analysis, we identified 13 key targets, which may be the key targets of EA anti‐GC, namely TP53, JUN, CASP3, HSP90AA1, VEGFA, HRAS, CDH1, MAPK3, CDKN1A, SRC, CYCS, BCL2L1 and CDK4. Among them, tumour protein p53 (TP53), a well‐known tumour suppressor gene, is the most frequently mutated gene in GC, accounting for about 50%.^50^ TP53 can induce cell cycle arrest, apoptosis and senescence, and this gene mutation plays an important role in the occurrence and development of GC.^51^ TP53 mutation is often used as a biomarker for diagnosis and prognosis, and as a potential therapeutic target for GC. CDKN1A (p21), cyclin‐dependent kinase inhibitor 1A, is a mediator of p53 tumour suppressor activity based on growth arrest, differentiation and senescence functions^52^; CDK4 is a member of the Ser/Thr protein kinase family, and its partner gene CDK6, is the core driver of the cell cycle^53^ and plays a crucial role in the occurrence and development of various malignant tumours including GC; p21 can promote tumorigenesis by promoting the assembly of CDK4/CDK6 complex.^54^ CDH1 (E‐cadherin) and its related signalling pathways play important physiological roles in maintaining cell adhesion, structure, motility and cell homeostasis^55^; CDH1 mutation is also associated with GC; and both dysregulation and mutation of the CDH1 gene can increase the proliferation, invasion and/or metastasis of GC cells and promote the occurrence and development of GC.^56^, ^57^ Transcription factor c‐Jun is a transcription factor with oncogenic function; c‐Jun is overexpressed in many cancers and plays an important role in various GC‐related pathological features such as apoptosis, proliferation, invasion, migration and drug resistance.^58^, ^59^, ^60^ CASP3 belongs to the Caspase protein family, an apoptotic pathway effector cysteine protease, which plays a central role in the execution stage of apoptosis and is a key gene commonly used to evaluate the effect of cancer therapy.^61^ Studies have shown that the expression of the key gene HSP90AA1 is closely related to the malignant phenotype of GC^62^ and is up‐regulated at the transcriptional and translational levels in primary GC compared with the normal gastric mucosa. Tumour angiogenesis is a key factor in the occurrence, development and metastasis of GC. VEGFA is an important pro‐angiogenic factor. Inhibiting VEGFA activity can inhibit the tumour growth and tumour angiogenesis of GC. It has become a common treatment strategy for many cancers including GC.^63^, ^64^ MAPK3 is a member of the MAPK family, which regulates various cellular processes such as cell proliferation, differentiation and cell cycle progression, and is implicated in the pathogenesis of various diseases.^65^ Src, a serine/threonine kinase, is often overexpressed or activated during GC development compared to normal tissues^66^, ^67^; Src activation can promote tumour development, tumour cell proliferation, migration and invasion.^68^, ^69^ BCL2L1 is an important anti‐apoptotic gene that is a member of the anti‐apoptotic Bcl‐2 protein family^70^ and is also a novel oncogenic driver in the progression of GC, which can be used as a potential candidate target for GC therapeutic drugs.^71^ In conclusion, the key targets predicted in this study are mainly related to the processes of tumour angiogenesis, apoptosis, proliferation, invasion and migration, and drug resistance, which preliminarily confirmed the possibility of EA against GC at the molecular level.

We conducted molecular docking between EA and 13 key targets, and the results showed that the binding energy of EA to these key targets was all less than −5.0 kcal/mol, and EA could effectively bind to key targets. Then, we took the protein–ligand complexes of EA and HSP90AA1, EA and MAPK3, EA and SRC with the highest binding energy as examples to carry out 10 ns MDS. By calculating RMSD and the results showed that EA formed stable protein–ligand complexes with HSP90AA1, MAPK3 and SRC. MM‐PBSA was used to calculate the binding free energy of the three protein–ligand complexes to provide more accurate affinity evaluation, the results showed that EA had good binding force with HSP90AA1, MAPK3, and SRC, and could form stable complexes. Above, through molecular docking and MDS, the accuracy of the predicted targets has been preliminarily verified, and EA may exert anticancer properties on GC by regulating these key targets.

The results of the KEGG pathway enrichment analysis showed that 13 key target genes were mainly enriched in Apoptosis, p53 signalling pathway and PI3K‐Akt signalling pathway. Apoptosis is an evolutionarily conserved program of cell death, and the activation of apoptotic pathways and related molecular targets is an important anticancer strategy^72^, ^73^; mammalian apoptosis pathway mainly includes extrinsic apoptosis pathway (receptor‐mediated) and intrinsic apoptosis pathway (mitochondria‐mediated), p53 signalling pathway and PI3K/AKT signalling pathway play an important role in this process. The p53 signalling pathway can induce cell cycle arrest, repair, senescence and apoptosis by regulating genes such as p53^74^; according to existing literature reports,^75^, ^76^ the p53 pathway is affected, which can promote the proliferation of GC cells, inhibit apoptosis and lead to cell cycle arrest, which can lead to the increase of GC malignant tumours. EA acts on TP53, CDKN1A, CDK4, BCL2L1, CYCS and CASP3, activates the p53 signalling pathway and induces cell cycle arrest, cell senescence and apoptosis to exert anticancer effects. The PI3K/AKT signalling pathway is an important growth regulatory pathway that regulates multiple physiological functions, including cell proliferation, differentiation, cell metabolism, apoptosis and cancer cell survival^77^; it has become a hot spot for tumour molecular marker‐targeted therapy,^78^, ^79^ more and more evidence^80^, ^81^ that the PI3K/Akt pathway is frequently activated in GC and is directly related to the occurrence and development of GC, and inhibition of the PI3K/Akt signalling pathway has been used as a therapeutic strategy for the treatment of GC patients. Yu's research showed^82^ that by inhibiting the PI3K/Akt pathway, GC cell proliferation and apoptosis can be effectively inhibited; in addition, Liu's team found that EA inhibited the growth and proliferation of non‐small cell lung cancer cells by downregulating the PI3K/Akt pathway^83^ and induced apoptosis by regulating the expression of apoptosis‐related proteins Bax, Bcl‐2 and caspase‐3 through this pathway. EA acts on VEGFA, HRAS, MAPK3, HSP90AA1, CDKN1A, CDK4, BCL2L1 and TP53, and regulates PI3K/AKT signalling pathway and its related cascade signalling pathways, participates in the inhibition of tumour‐related angiogenesis, DNA repair, cell cycle, and induces apoptosis and other related mechanisms to play an anti‐GC role.

Although this study provides evidence for the potential effect of EA against GC, however, it should be mentioned that this study still has certain limitations. The relevant targets are obtained from the database, which may depend on the accuracy of the database. Further in vitro and in vivo experimental verification is required to better develop EA for clinical application.

## 5 CONCLUSION

In conclusion, this study adopted bioinformatics analysis and network pharmacology revealed that EA anti‐GC mainly regulated TP53, JUN, CASP3, HSP90AA1, VEGFA, HRAS, CDH1, MAPK3, CDKN1A, SRC, CYCS, BCL2L1 and CDK4 as well as p53 signalling pathway, PI3K‐Akt signalling pathway, involved in the inhibition of tumour angiogenesis, cell cycle, cell proliferation, invasion and migration and induction of apoptosis. It reveals that EA has great potential for the prevention and treatment of GC, and provides a theoretical basis for the further study of EA against GC.

## AUTHOR CONTRIBUTIONS

**Zhiyao Liu:** Conceptualization (equal); methodology (equal); writing – original draft (equal). **Hailiang Huang:** Funding acquisition (equal); writing – review and editing (equal). **Ying Yu:** Data curation (equal); software (equal). **Lingling Li:** Data curation (equal); software (equal). **Xin Shi:** Validation (equal). **Fangqi Wang:** Validation (equal).

## FUNDING INFORMATION

This study was funded by the High Level Key Disciplines of Traditional Chinese Medicine Basic Theory of Traditional Chinese Medicine, National Administration of Traditional Chinese. Medicine, Shandong University of Traditional Chinese Medicine, Jinan 250355, PR China and the Key Laboratory of Traditional Chinese Medicine Classical Theory, Ministry of Education, Shandong University of Traditional Chinese Medicine, Jinan 250355, PR China.

## CONFLICT OF INTEREST STATEMENT

The authors declare that there are no competing interests associated with the manuscript.

## Data Availability

The data used to support the findings of this study are available from the corresponding author upon request.
